# The Clínica Universidad de Navarra-Body Adiposity Estimator index is a reliable tool for prediabetes: a multi-center retrospective cohort study

**DOI:** 10.3389/fnut.2025.1685618

**Published:** 2025-10-24

**Authors:** Fuyi Ma, Ruwen Wang, Ru Wang

**Affiliations:** School of Exercise and Health, Shanghai University of Sport, Shanghai, China

**Keywords:** prediabetes, CUN-BAE, Chinese adults, BMI, body fat

## Abstract

**Objective:**

Given the close link between body fat and insulin resistance, our study aimed to evaluate the association and predictive value of the Clínica Universidad de Navarra Body Adiposity Estimator (CUN-BAE) in identifying the risk of prediabetes.

**Methods:**

This multi-center retrospective cohort study included 112,708 participants from 32 regions across 11 cities in China. Multivariable Cox regression and restricted cubic spline (RCS) analyses were used to assess the association between CUN-BAE and prediabetes risk. Predictive effectiveness was evaluated using receiver operating characteristic (ROC) curves.

**Results:**

An independent association of CUN-BAE with prediabetes was significantly shown after adjusting for important covariates. CUN-BAE is nonlinearly positively correlated with prediabetes. In the male subgroup analysis, higher triglyceride levels or hypertension in combination with CUN-BAE significantly increased the incidence of prediabetes (interaction *p* < 0.05 for both). In the female subgroup analysis, middle age, BMI > 28, higher triglyceride levels, or hypertension in combination with CUN-BAE significantly increased the incidence of prediabetes (interaction *p* < 0.05 for all). ROC analysis demonstrated that CUN-BAE performed better in predicting risk than BMI, TG/HDL-c and TyG.

**Conclusion:**

The CUN-BAE index was independently and nonlinearly positively associated with an increased risk of prediabetes and exhibited high predictive accuracy.

## 1 Introduction

Prediabetes is an increasingly serious threat to global health (1). As of 2021, an estimated 762 million people globally have prediabetes, and this number is projected to increase to 1.052 billion by 2045 (2). In 2018, a nationally representative cross-sectional study in China reported a 38.1% prevalence of prediabetes (3). Without timely intervention, the annual conversion rate from prediabetes to diabetes ranges from 5 to 10%, leading to a cumulative diabetes incidence of 95.9% after 30 years in China (4, 5). Prediabetes not only increases the risk of developing diabetes but also elevates the risk of future cardiovascular diseases, dementia, depression, cancer, and other conditions (6–9). Therefore, the detection and intervention of prediabetes are critical for preventing diabetes and its associated complications.

In individuals with overweight or obesity, excessive adipose tissue accumulation induces insulin resistance, thereby accelerating the initiation and progression of prediabetes (10–13). While Body Mass Index (BMI), a widely used metric, provides a convenient method for differentiating between overweight and obese individuals (14, 15). However, this traditional indicator lacks the ability to accurately characterize muscle and fat composition and is confounded by sex and age, factors that collectively compromise its precision in in obesity evaluation (16–18). Consequently, reliable methods for direct or indirect assessment of body fat content are essential for effective obesity evaluation. The body fat percentage requires special instruments such as Dual-Energy X-ray Absorptiometry (DEXA) and air displacement plethysmography, but their clinical application may be limited owing to the high cost and time requirements (19, 20). To address these limitations, in 2011, Professor Javier Gómez-Ambrosi developed the Clínica Universidad de Navarra Body Adiposity Estimator (CUN-BAE), a new equation for estimating body fat (21). This method estimates body fat percentage (BF%) based on BMI, gender, and age derived from the data of 6,510 participants. This study showed that CUN-BAE is highly effective in estimating BF%, with a strong correlation (*r* = 0.89) between CUN-BAE and actual BF% measured by air displacement plethysmography, surpassing other common anthropometric indicators. Recent research has further validated the CUN-BAE index as a robust tool for identifying various health conditions. For example, a large-scale analysis involving 12,328 Polish individuals revealed that the CUN-BAE outperformed six other anthropometric measures in predicting the risk of metabolic syndrome among males (22). In another study that included 81,532 elderly Chinese participants, CUN-BAE exhibited a stronger correlation with cardiometabolic multimorbidity than traditional obesity indicators such as BMI. This underscores its effectiveness in identifying high-risk individuals within this demographic (23). Additionally, CUN-BAE has shown strong predictive performance for hypertension events (24). Overall, these findings highlight the strong correlation and predictive power of CUN-BAE with a number of metabolic diseases.

Despite these findings, research on the relationship between CUN-BAE index and prediabetes risk remains limited, highlighting the need for further investigation through large-scale longitudinal studies. In this study, we used data from a national representative sample to explore the relationship between the CUN-BAE index and prediabetes risk in Chinese adults.

## 2 Materials and methods

### 2.1 Data sources and study population

All the data used in this study were obtained from the public database Dryad,^1^ with the original data provided by Chen et al. (25). This database includes the medical records of Chinese adults over 20 years of age (*n* = 685,227) who underwent health screenings at Rich Healthcare Group health examination centers across 32 districts in 11 cities and at least two health examinations between 2010 and 2016. According to the Dryad database terms, researchers are permitted to use these data for secondary analysis.

**Figure 1:**
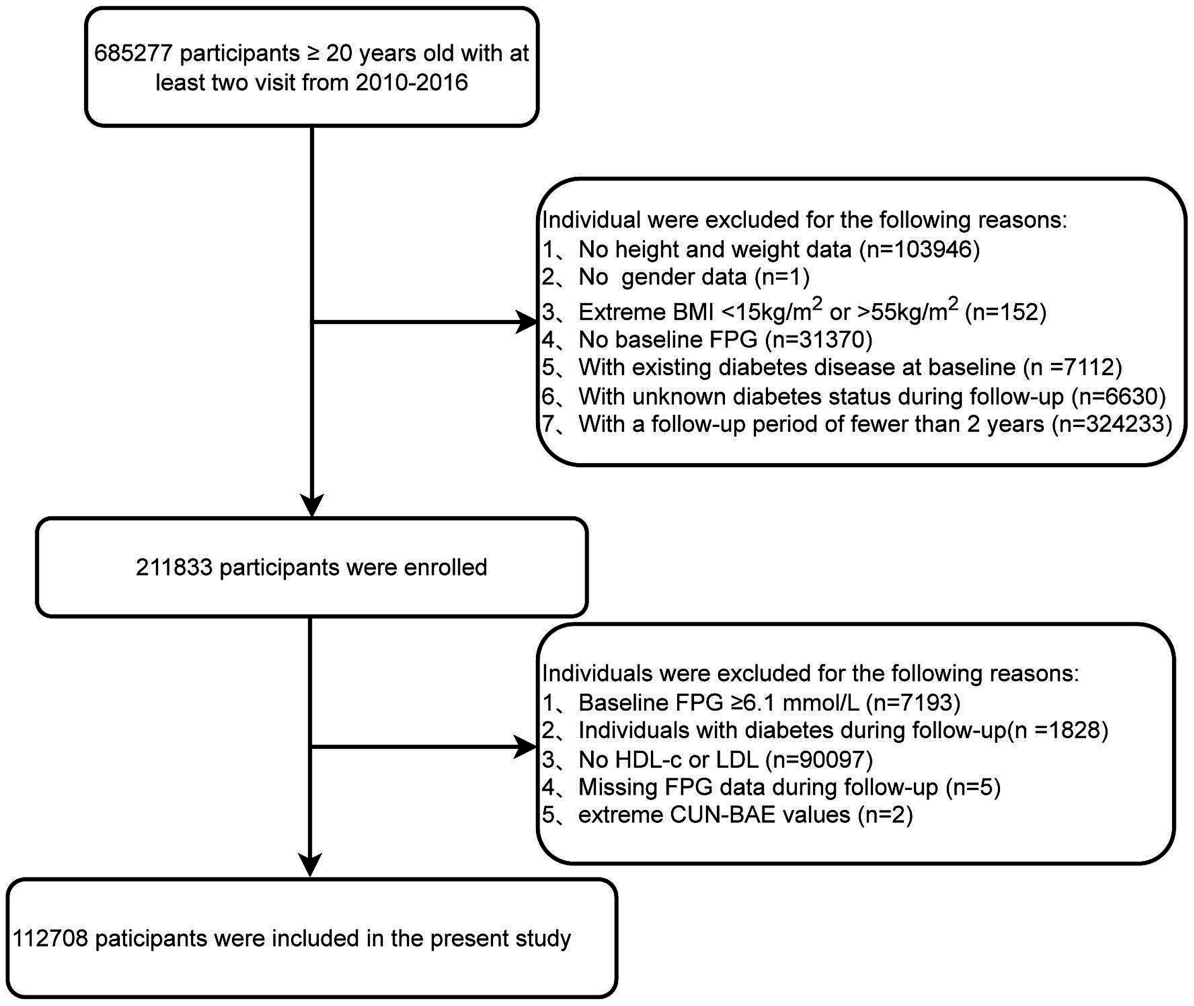
Flowchart of the study population.

We excluded the following participants: (1) those with missing height and weight data (*n* = 103,946); (2) those with unknown gender (*n* = 1); (3) those with abnormal BMI values, specifically <15 or >55 kg/m^2^ (*n* = 152); (4) those with missing baseline fasting blood glucose data (*n* = 31,370); (5) those diagnosed with diabetes at baseline (*n* = 7,112); (6) those with unknown diabetes status during follow-up (*n* = 6,630); (7) those with a follow-up period of less than 2 years (*n* = 324,233). We instituted this minimum follow-up requirement to ensure sufficient time for the identification of new-onset prediabetes, which is a gradual metabolic process (26). A shorter follow-up interval might not adequately capture the longitudinal changes in fasting plasma glucose and could lead to an underestimation of the true incidence. To ensure a baseline cohort of participants with normal glucose metabolism, we excluded individuals with prediabetes or diabetes at baseline using the WHO criteria (27). Specifically, we excluded participants with: (1) baseline fasting blood glucose ≥6.1 mmol/L (*n* = 7,193); (2) individuals with diabetes during follow-up (*n* = 1,828); (3) missing HDL-c or LDL-c values (*n* = 90,097); (4) missing follow-up fasting plasma glucose data (*n* = 5); (5) abnormal CUN-BAE values (more than three standard deviations above or below the mean) (*n* = 2). The final analysis included 112,708 eligible participants (Figure 1).

### 2.2 Data collection

Upon arrival at the health examination center, the participants completed a detailed questionnaire. The questionnaire gathered information on age, gender, smoking and drinking habits, and family history of diabetes. Height and weight were measured by the staff, with participants removing heavy clothing and shoes to ensure accuracy. Trained personnel measured blood pressure using a standard mercury sphygmomanometer. In addition, the indices were calculated using the following formulas: BMI was calculated as weight (kg) divided by height in meters squared (m^2^), TG/HDL-c = TG (mg/dL)/ HDL-c (mg/dL), TyG = ln [triglycerides (mg/dL) × glucose (mg/dL)/2] (28).

After fasting for at least 10 h, venous blood samples were collected by specialized medical staff. An automated analyzer (Beckman Coulter AU5800, Brea, CA, USA) was used to measure triglycerides (TG), total cholesterol (TC), high-density lipoprotein cholesterol (HDL-c), low-density lipoprotein cholesterol (LDL-c), alanine aminotransferase (ALT), aspartate aminotransferase (AST), creatinine (Cr), fasting plasma glucose (FPG), and blood urea nitrogen (BUN). Fasting blood glucose levels were measured using the glucose oxidase method, whereas other indicators were determined using the optical turbidimetric method.

### 2.3 Diagnosis of prediabetes

Incident prediabetes was defined according to the World Health Organization (WHO) criteria (27). Participants were ascertained to have developed incident prediabetes if they had a fasting plasma glucose level between 6.1 and 6.9 mmol/L at any follow-up examination.

### 2.4 The calculation of CUN-BAE

The new body fat index, CUN-BAE, used to assess the percentage of body fat was calculated as: CUN-BAE = −44.988 + (0.503 × age) + (10.689 × sex) + (3.172 × BMI) − (0.026 × BMI^2^) + (0.181 × BMI × sex) − (0.02 × BMI × age) − (0.005 × BMI^2^ × sex) + (0.00021 × BMI^2^ × age), where male is 0, female is 1, and age is in years (21).

### 2.5 Statistical analysis

All data analyses were conducted using R software (version 4.4.1). Statistical significance was set at a two-sided *p*-value <0.05. We tested this hypothesis through a series of rigorous analytical steps.

First, to maximize the sample size, we applied multiple imputations to handle missing data, despite the small proportion of missing values (<10%) (Supplementary Figure S1). We used Multiple Imputation by Chained Equations and performed five imputations to minimize estimation errors. We then validated the imputed data to ensure consistency with the original data distribution and statistical characteristics, thus minimizing the impact of missing data treatment on the results.

Second, the participants were divided into four groups based on the CUN-BAE quartiles (Q1 to Q4), and their baseline characteristics were presented. Continuous variables are expressed as mean ± standard deviation and group differences were tested using one-way analysis of variance. Categorical variables were presented as counts (percentages), and group differences were analyzed using Pearson’s chi-square test.

Next, we calculated survival curves using the Kaplan–Meier method to determine the cumulative incidence of prediabetes, defined as the first occurrence of fasting blood glucose levels between 6.1 and 6.9 mmol/L. The log-rank test was used to compare differences between CUN-BAE quartiles. The time-to-event was defined as the interval from the baseline examination to either the first diagnosis of prediabetes (event) or the last available examination. The follow-up time was treated as a continuous variable in years, derived from the exact dates of the health examinations. Multivariable Cox regression models were used to estimate hazard ratios (HRs) and 95% confidence intervals (CIs) for the association between CUN-BAE and prediabetes risk, adjusted for covariates. Model 1 was adjusted for age, height, and family history of diabetes. Model 2 included additional adjustments for systolic and diastolic blood pressure. Model 3 was further adjusted for HDL-c, LDL-c, ALT, BUN, Cr, TG and TC levels, along with all covariates from Model 2. To describe the nonlinear and dose–response relationship between CUN-BAE and prediabetes risk in more detail, we applied a fully adjusted RCS model. The optimal number of knots (five for male and four for female) was selected based on the lowest Akaike Information Criterion (AIC) to achieve the best model fit while avoiding overfitting. Additionally, we conducted subgroup and interaction analyses based on Model 3 to explore other factors that influence the relationship between CUN-BAE and prediabetes risk. These analyses were stratified by age (≤45 years, 45–65 years, >65 years), BMI (≤23.9, 24.0–27.9, ≥28), TG (<1.7 mmol/L, ≥1.7 mmol/L), TC (<5.2 mmol/L, ≥5.2 mmol/L), and Hypertension (No, Yes).

Subsequently, we constructed ROC curves and adjusted for variables such as height, family history, systolic blood pressure (SBP), diastolic blood pressure (DBP), HDL, LDL, ALT, BUN, CR, TG and TC to evaluate the effectiveness of CUN-BAE and BMI in predicting the risk of prediabetes. The areas under the ROC curves were compared using the DeLong test to identify the optimal predictor risk of prediabetes.

Finally, to ensure the robustness of the results, we conducted a sensitivity analysis. First, we excluded participants with missing variable values to eliminate the potential impact of missing data on outcomes and refitted the univariate and multivariate Cox regression models. Second, we created a well-matched cohort (Q1–Q2 vs. Q3–Q4) using 1:1 propensity score matching with nearest neighbor matching without replacement and a caliper width of 0.2. We used a standardized mean difference (SMD) of less than 0.10 to ensure balance between groups. Third, we redefined the prediabetes status according to the American Diabetes Association (ADA) criteria and included this in the analysis of the study subjects. Lastly, we further incorporated smoking and alcohol consumption variables into the multivariate Cox regression models to evaluate their potential impact on the outcomes.

## 3 Results

### 3.1 Baseline characteristics of participants

Following the World Health Organization’s diagnostic criteria for prediabetes, the final analysis included 112,708 participants with normal blood glucose levels. A comparison with the excluded cohort revealed no clinically significant differences (all SMD < 0.2, Supplementary Table S1), suggesting a low risk of selection bias. The included cohort comprised 59,918 males (53.2%) with a mean (±SD) age of 43.64 ± 12.75 years (Supplementary Table S2).

We categorized participants’ baseline characteristics by gender and further classified them according to CUN-BAE quartiles, as summarized in Table 1. The average CUN-BAE (SD) was 22.65 ± 5.19 for male and 31.44 ± 5.41 for female. Lower HDL-c levels were observed in individuals in the higher quartile groups (Q2 to Q4) compared to those in the lowest quartile group (Q1) in both male and female groups. These individuals were also younger and had higher levels of SBP, DBP, FPG, TC, TG, BUN, LDL-c, Cr, and ALT.

**Table 1:** Baseline characteristics of study participants by gender according to CUN-BAE index quartiles.

Characteristics	Overall	Quartiles of the CUN-BAE				*p*-value
		Quartile 1	Quartile 2	Quartile 3	Quartile 4	
Male						
*n*	59,918	14,981	14,992	14,957	14,988	
Age, year	43.89 ± 13.01	35.40 ± 7.97	41.73 ± 10.63	46.96 ± 12.39	51.47 ± 14.25	<0.001
Height, cm	171.73 ± 6.23	172.85 ± 6.07	172.04 ± 6.11	171.32 ± 6.12	170.69 ± 6.40	<0.001
BMI, kg/m^2^	24.24 ± 3.15	20.68 ± 1.57	23.29 ± 1.26	25.03 ± 1.38	27.99 ± 2.32	<0.001
Smoking status						<0.001
Current	6,253 (30.85)	1,234 (23.34)	1,512 (29.21)	1720 (34.59)	1787 (36.97)	
Former	1,252 (6.18)	286 (5.41)	331 (6.39)	328 (6.60)	307 (6.35)	
Never	12,765 (62.97)	3,767 (71.25)	3,334 (64.40)	2,924 (58.81)	2,740 (56.68)	
Drinking status						<0.001
Current	781 (3.85)	109 (2.06)	183 (3.53)	223 (4.49)	266 (5.50)	
Former	4,987 (24.60)	1,171 (22.15)	1,369 (26.44)	1,293 (26.01)	1,154 (23.87)	
Never	14,502 (71.54)	4,007 (75.79)	3,625 (70.02)	3,456 (69.51)	3,414 (70.62)	
Family history						<0.001
Yes	58,947 (98.38)	14,786 (98.70)	14,711 (98.13)	14,700 (98.28)	14,750 (98.41)	
No	971 (1.62)	195 (1.30)	281 (1.87)	257 (1.72)	238 (1.59)	
SBP, mmHg	122.54 ± 15.50	116.68 ± 13.03	119.95 ± 13.92	123.76 ± 15.24	129.76 ± 16.47	<0.001
DBP, mmHg	76.70 ± 10.67	72.25 ± 8.98	75.25 ± 9.74	77.94 ± 10.38	81.36 ± 11.27	<0.001
FPG, mmol/L	4.92 ± 0.56	4.79 ± 0.55	4.88 ± 0.55	4.97 ± 0.55	5.06 ± 0.55	<0.001
TC, mmol/L	4.79 ± 0.88	4.47 ± 0.80	4.77 ± 0.85	4.91 ± 0.88	5.00 ± 0.89	<0.001
TG, mg/dL	1.59 ± 1.14	1.10 ± 0.64	1.50 ± 1.00	1.75 ± 1.16	2.03 ± 1.40	<0.001
BUN, mmol/L	4.92 ± 1.15	4.78 ± 1.11	4.86 ± 1.12	4.97 ± 1.17	5.06 ± 1.18	<0.001
HDL-c, mmol/L	1.29 ± 0.28	1.36 ± 0.27	1.30 ± 0.27	1.27 ± 0.27	1.23 ± 0.27	<0.001
LDL-c, mg/dL	2.78 ± 0.67	2.58 ± 0.61	2.79 ± 0.65	2.87 ± 0.68	2.90 ± 0.69	<0.001
CR, μmol/L	80.61 ± 11.88	79.92 ± 11.08	80.30 ± 11.13	80.81 ± 12.05	81.42 ± 13.08	<0.001
ALT, U/L	29.13 ± 23.52	21.99 ± 18.27	27.52 ± 20.86	31.02 ± 25.44	36.00 ± 26.30	<0.001
CUN-BAE	22.65 ± 5.19	15.85 ± 2.70	21.19 ± 1.03	24.52 ± 0.96	29.04 ± 2.39	<0.001
Female						
*n*	52,790	13,182	13,215	13,186	13,207	
Age, year	43.35 ± 12.45	33.29 ± 5.52	39.53 ± 8.40	46.35 ± 10.70	54.21 ± 12.70	<0.001
Height, cm	160.10 ± 5.65	161.72 ± 5.35	160.75 ± 5.41	159.71 ± 5.47	158.22 ± 5.77	<0.001
BMI, kg/m^2^	22.14 ± 3.03	18.92 ± 1.12	20.98 ± 1.03	22.67 ± 1.27	25.99 ± 2.45	<0.001
Smoking status						0.49
Current	17 (0.15)	6 (0.20)	2 (0.07)	4 (0.15)	5 (0.19)	
Former	11 (0.10)	2 (0.07)	1 (0.04)	5 (0.18)	3 (0.11)	
Never	11,137 (99.75)	2,980 (99.73)	2,813 (99.89)	2,708 (99.67)	2,636 (99.70)	
Drinking status						0.16
Current	17(0.15)	5 (0.17)	4 (0.14)	6 (0.22)	2 (0.08)	
Former	305(2.73)	66 (2.21)	72 (2.56)	79 (2.91)	88 (3.33)	
Never	10,843 (97.12)	2,917 (97.62)	2,740(97.30)	2,632(96.87)	2,554 (96.60)	
Family history						<0.001
Yes	51,230 (97.04)	12,880 (97.71)	12,793 (96.81)	12,711 (96.40)	12,846 (97.27)	
No	1,560 (2.96)	302 (2.29)	422 (3.19)	475 (3.60)	361 (2.73)	
SBP, mmHg	114.86 ± 16.53	107.37 ± 11.60	109.99 ± 12.97	115.72 ± 15.39	126.34 ± 18.46	<0.001
DBP, mmHg	71.36 ± 10.43	67.65 ± 8.48	68.94 ± 9.24	71.98 ± 10.13	76.86 ± 11.19	<0.001
FPG, mmol/L	4.84 ± 0.53	4.68 ± 0.51	4.78 ± 0.50	4.88 ± 0.52	5.02 ± 0.52	<0.001
TC, mmol/L	4.76 ± 0.91	4.43 ± 0.77	4.62 ± 0.84	4.85 ± 0.90	5.13 ± 0.96	<0.001
TG, mg/dL	1.07 ± 0.72	0.77 ± 0.38	0.89 ± 0.51	1.12 ± 0.71	1.50 ± 0.94	<0.001
BUN, mmol/L	4.37 ± 1.13	4.12 ± 1.02	4.23 ± 1.06	4.42 ± 1.11	4.71 ± 1.21	<0.001
HDL-c, mmol/L	1.47 ± 0.31	1.54 ± 0.31	1.50 ± 0.31	1.45 ± 0.31	1.40 ± 0.29	<0.001
LDL-c, mg/dL	2.73 ± 0.69	2.49 ± 0.58	2.64 ± 0.63	2.81 ± 0.68	3.00 ± 0.73	<0.001
CR, umol/L	58.30 ± 10.35	57.29 ± 8.69	57.58 ± 9.98	58.32 ± 9.53	60.02 ± 12.57	<0.001
ALT, U/L	17.18 ± 17.20	13.88 ± 11.48	15.37 ± 15.99	17.69 ± 21.62	21.79 ± 17.12	<0.001
CUN-BAE	31.44 ± 5.41	24.67 ± 2.12	29.38 ± 1.09	33.15 ± 1.13	38.54 ± 2.56	<0.001

In addition, the distribution of CUN-BAE was normally distributed across genders (Supplementary Figure S2).

### 3.2 The relationship between CUN-BAE and the incidence of prediabetes

**Figure 2:**
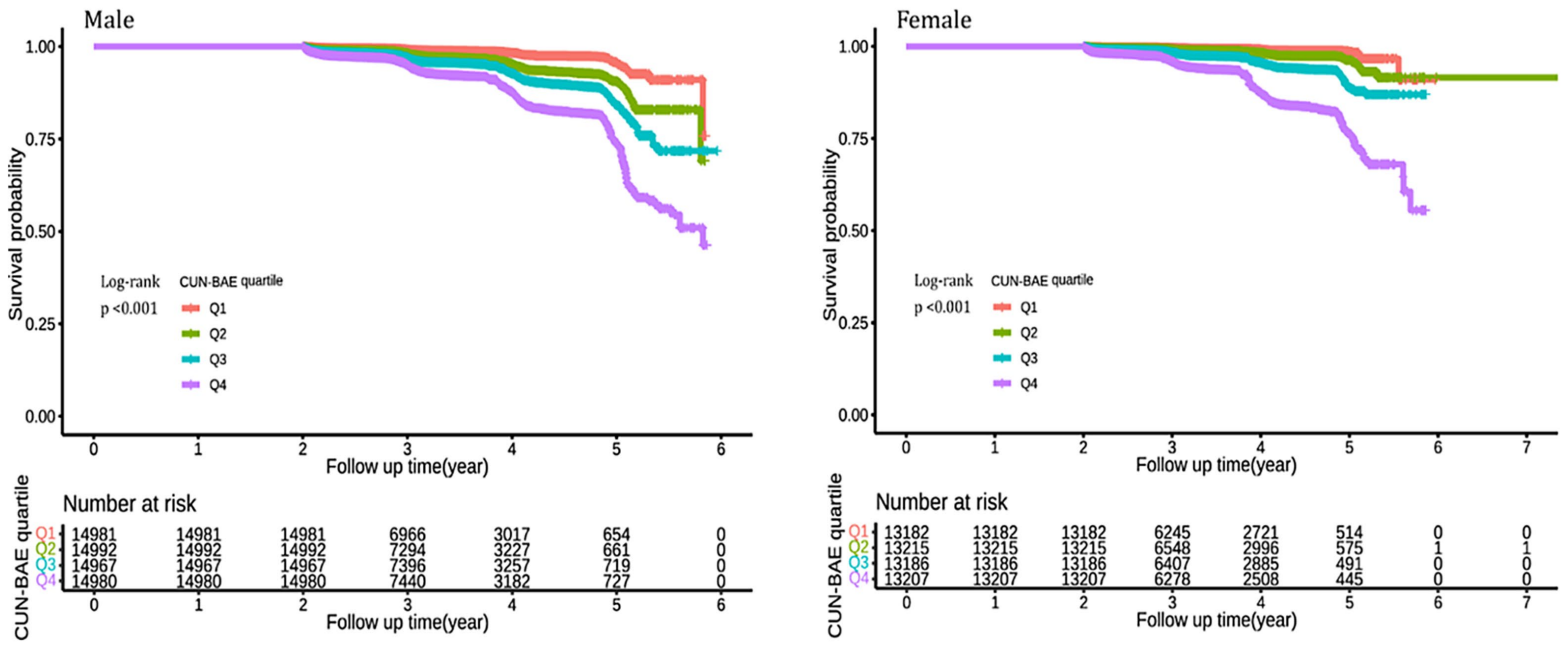
Kaplan–Meier analysis of future prediabetes risk by according to CUN-BAE quartile among male and female. The Kaplan–Meier curves illustrate the cumulative incidence of prediabetes over time in male and female participants, categorized by quartiles (Q1–Q4) of the baseline CUN-BAE index. Differences between curves were assessed using the log-rank test, and *p*-values <0.001 for both genders indicate statistically significant separation between the quartile groups.

We found that higher CUN-BAE levels were associated with an increased incidence of prediabetes in both male and female groups. Over an average follow-up period of 3.11 years, 4,125 participants (3.66%) were newly diagnosed with prediabetes. Of these, 2,623 (4.38%) were male, and 1,502 (2.85%) were female. As shown in Supplementary Figure S3, the incidence of prediabetes progressively increased across CUN-BAE quartiles (Q1 to Q4) for both male and female. In the male group, the incidence rates were 169 cases (1.13%) in Q1, 450 cases (3.00%) in Q2, 719 cases (4.81%) in Q3, and 1,285 cases (8.57%) in Q4. In the female group, the incidence rates were 65 cases (0.49%) in Q1, 153 cases (1.16%) in Q2, 363 cases (2.75%) in Q3, and 921 cases (6.97%) in Q4. As illustrated in Figure 2, the cumulative incidence of prediabetes increased with higher CUN-BAE quartiles in both male and female groups (log-rank *p* < 0.001).

After adjusting for multiple covariates, the Cox regression model revealed that higher CUN-BAE levels (Q2 to Q4) were significantly associated with an increased risk of prediabetes in the male group [HR, 95% CI: 1.88 (1.57–2.25) for Q2; 2.37 (1.98–2.83) for Q3; 3.19 (2.66–3.81) for Q4] (Table 2). When analyzed as a continuous variable, each standard deviation increase in CUN-BAE was significantly associated with a higher risk of prediabetes [HR, 95% CI: 1.47 (1.40–1.55)]. Similarly, in the female group, higher CUN-BAE levels (Q2 to Q4) were significantly associated with an increased risk of prediabetes [HR, 95% CI: 1.80 (1.35–2.41) for Q2; 3.24 (2.47–4.27) for Q3; 5.34 (4.03–7.07) for Q4]. When treated as a continuous variable, each standard deviation increase in CUN-BAE was also significantly associated with prediabetes events [HR, 95% CI: 1.65 (1.54–1.76)].

**Table 2:** Association between the baseline CUN-BAE index and incident prediabetes among male and female.

CUN-BAE	Crude model		Model 1		Model 2		Model 3	
	HR (95%CI)	*p*	HR (95%CI)	*p*	HR (95%CI)	*p*	HR (95%CI)	*p*
Male								
Quartiles								
Quartiles 1	Reference		Reference		Reference		Reference	
Quartiles 2	2.53 (2.12, 3.02)	<0.001	2.09 (1.75, 2.50)	<0.001	2.00 (1.67, 2.39)	<0.001	1.88 (1.57, 2.25)	<0.001
Quartiles 3	3.97 (3.36, 4.69)	<0.001	2.80 (2.36, 3.33)	<0.001	2.55 (2.15, 3.04)	<0.001	2.37 (1.98, 2.83)	<0.001
Quartiles 4	7.04 (6.00, 8.27)	<0.001	4.26 (3.59, 5.05)	<0.001	3.57 (3.00, 4.25)	<0.001	3.19 (2.66, 3.81)	<0.001
*p* for trend		<0.001		<0.001		<0.001		<0.001
Per 1 SD increase	1.96 (1.88, 2.04)	<0.001	1.66 (1.59, 1.75)	<0.001	1.54 (1.46, 1.62)	<0.001	1.47 (1.40, 1.55)	<0.001
Female								
Quartiles								
Quartiles 1	Reference		Reference		Reference		Reference	
Quartiles 2	2.21 (1.66, 2.96)	<0.001	1.76 (1.31, 2.35)	<0.001	1.82 (1.36, 2.43)	<0.001	1.80 (1.35, 2.41)	0.010
Quartiles 3	5.37 (4.12, 6.99)	<0.001	3.31 (2.52, 4.35)	<0.001	3.35 (2.55, 4.40)	<0.001	3.24 (2.47, 4.27)	<0.001
Quartiles 4	14.3 (11.12, 18.39)	<0.001	6.42 (4.88, 8.44)	<0.001	5.83 (4.42, 7.68)	<0.001	5.34 (4.03, 7.07)	<0.001
*p* for trend		<0.001		<0.001		<0.001		<0.001
Per 1 SD increase	2.3 (2.20, 2.41)	<0.001	1.83 (1.72, 1.94)	<0.001	1.7 (1.60, 1.81)	<0.001	1.65 (1.54, 1.76)	<0.001

**Figure 3:**
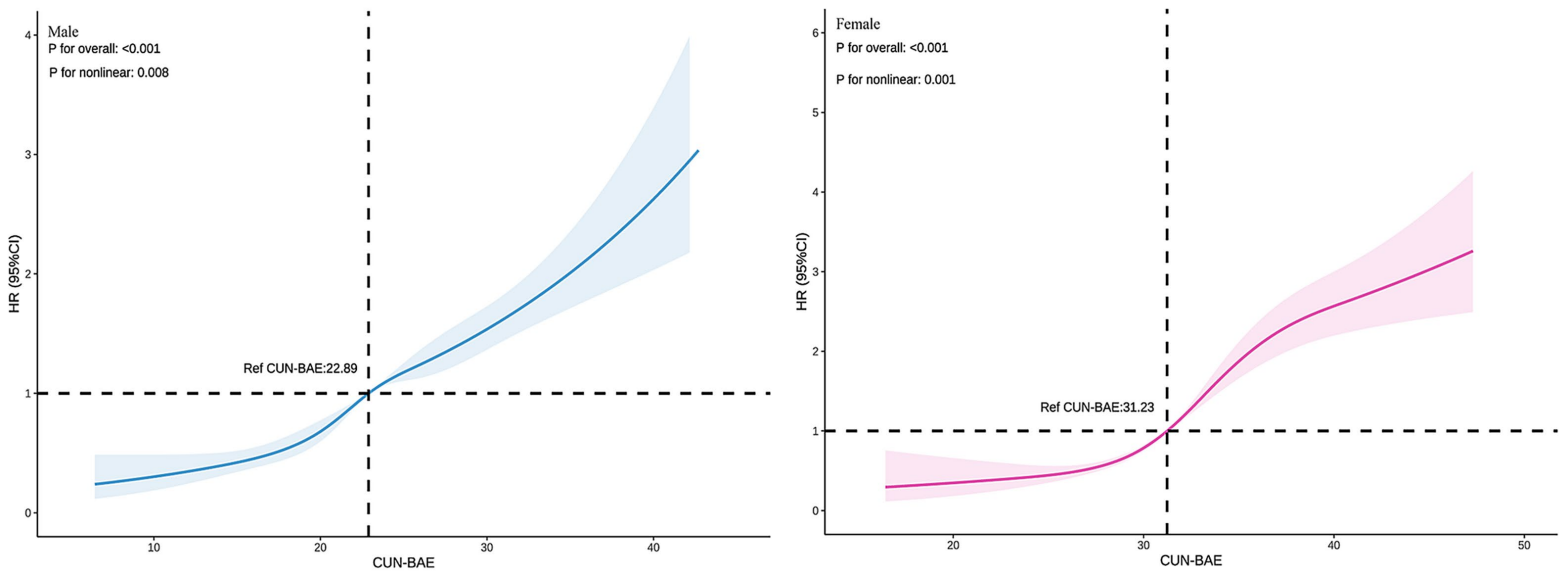
Association between the baseline CUN-BAE index and incident prediabetes among male and female. The RCS plots depict the nonlinear association of the continuous CUN-BAE index with the hazard ratio (HR) of incident prediabetes in male and female, based on multivariable Cox proportional hazards models (Model 3). The solid line represents the estimated HR, and the shaded area indicates the 95% confidence interval.

In both male and female groups, the fully adjusted RCS regression model indicated a nonlinear relationship between CUN-BAE and prediabetes risk (*p* for overall = 0.001, *p* for nonlinear = 0.008; *p* for overall = 0.001, *p* for nonlinear = 0.001, respectively), as detailed in Figure 3.

### 3.3 Subgroup analysis and interaction testing

**Figure 4:**
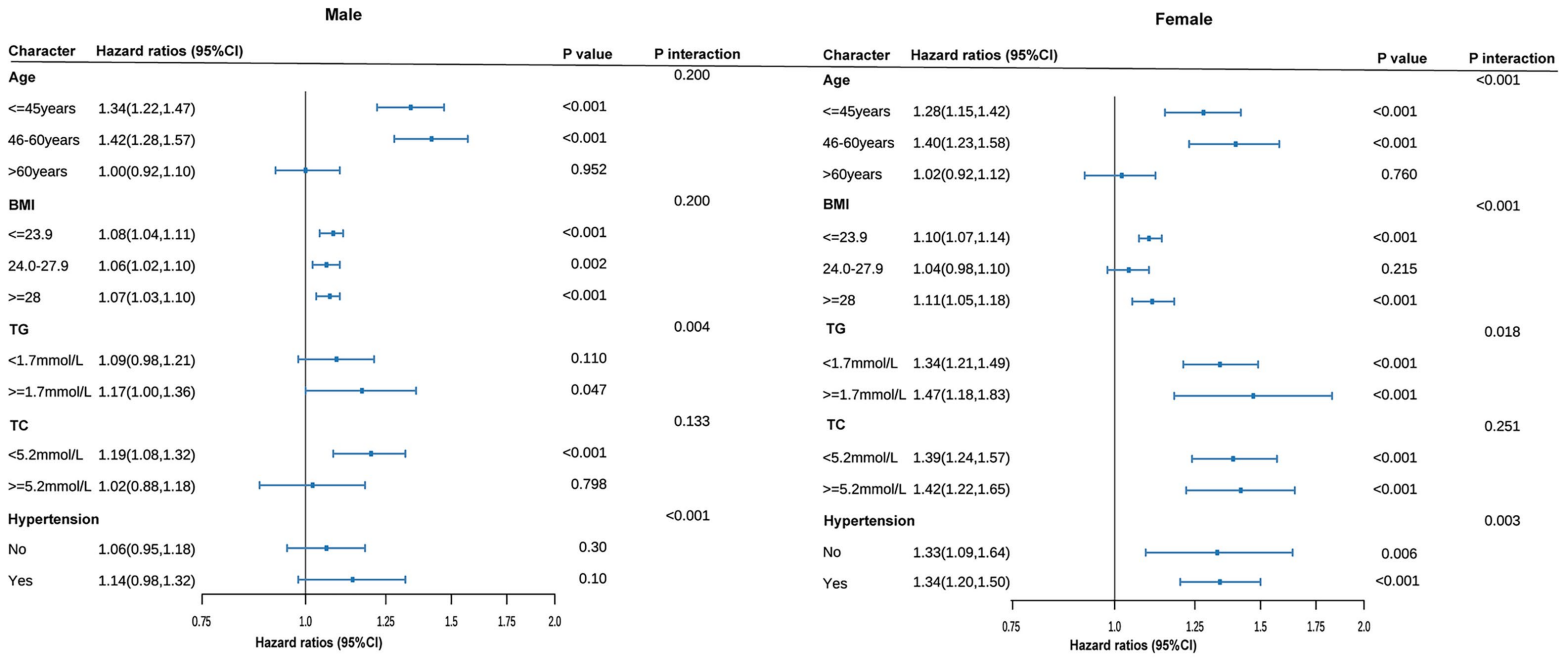
Subgroup analyses of association between the baseline CUN-BAE index and incident prediabetes among male and female. All models are adjusted for covariates in Model 3. The size of the data markers corresponds to the sample size of each subgroup. *p* for interaction represents the significance level of the interaction term between the subgroup variable and the continuous CUN-BAE index.

Our analysis reveals a significant relationship between CUN-BAE and the risk of prediabetes, with this association being particularly pronounced in specific subgroups. To explore this further, we conducted a series of subgroup analyses, which are detailed in Figure 4. These analyses, stratified by Age, BMI, TG, TC, and Hypertension, consistently demonstrated a significant association between elevated CUN-BAE levels and an increased risk of prediabetes across both male and female cohorts. Additionally, in males, individuals with higher TG levels or hypertension were found to have a significantly higher risk of prediabetes with increasing CUN-BAE (*p* interaction <0.05). In contrast, among females, middle-aged individuals with obesity (BMI ≥ 28), higher TG levels, or hypertension exhibited a stronger association between elevated CUN-BAE and prediabetes risk (*p* interaction <0.05).

### 3.4 The predictive value of CUN-BAE for prediabetes events

**Figure 5:**
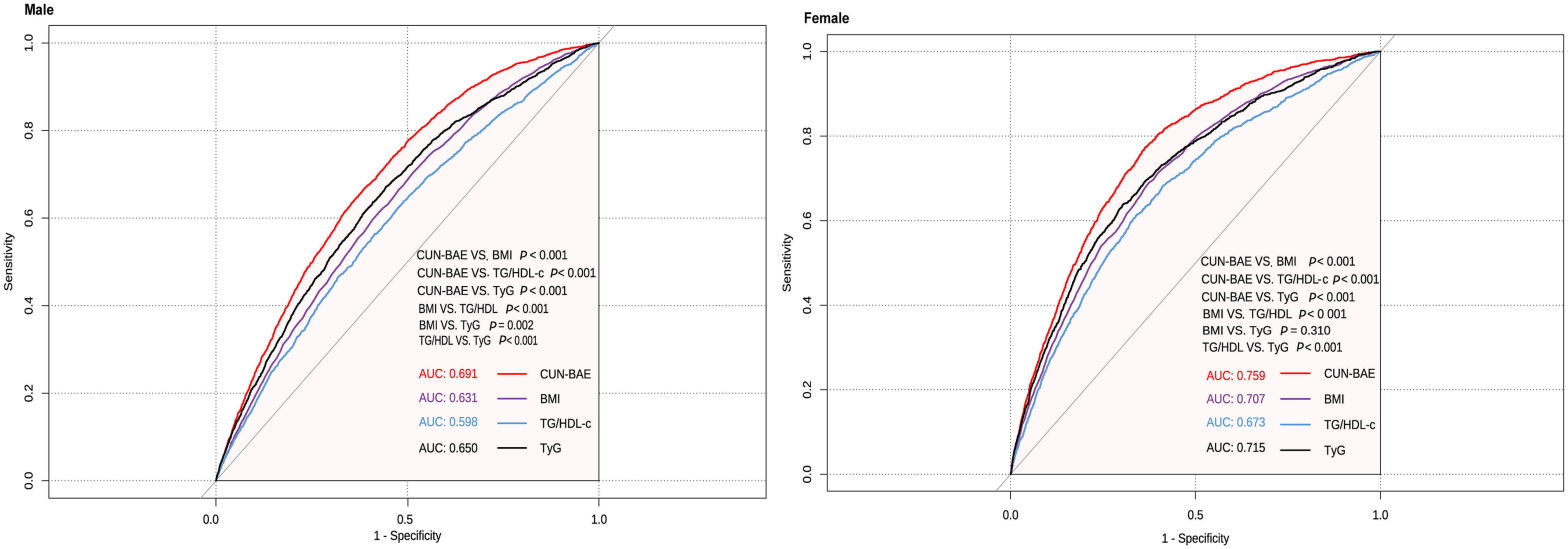
Areas under the receiver operating characteristic curves for CUN-BAE, BMI, TG/HDL-c, and TyG in identifying prediabetes among male and female.

Our analysis demonstrates that CUN-BAE exhibits superior accuracy in predicting prediabetes compared to BMI, TG/HDL-c, and TyG. A ROC curve analysis was performed to evaluate the accuracy of CUN-BAE, BMI, TG/HDL-c, and TyG in identifying prediabetes (Figure 5). The AUC values with 95% confidence intervals for each indicator are as follows: CUN-BAE (AUC: 0.691, 95% CI: 0.681–0.701), BMI (AUC: 0.631, 95% CI: 0.620–0.641), TG/HDL-c (AUC: 0.598, 95% CI: 0.587–0.609), TyG (AUC: 0.650, 95% CI: 0.639–0.660) in male; CUN-BAE (AUC: 0.759, 95% CI: 0.748–0.770), BMI (AUC: 0.707, 95% CI: 0.694–0.720), TG/HDL-c (AUC: 0.673, 95% CI: 0.659–0.687), TyG (AUC: 0.715, 95% CI: 0.702–0.728) in female. Pairwise comparisons using the DeLong test revealed that the AUC of CUN-BAE was significantly greater than that of all other indices in both males and females (all *p* < 0.001), confirming its stronger predictive capability.

### 3.5 Sensitivity analysis

The significant association between CUN-BAE and incident prediabetes proved highly robust across all sensitivity analyses. Hazard ratios remained statistically significant and of comparable magnitude in both sexes when: (1) using a complete-case analysis; (2) analyzing a propensity score-matched cohort; (3) applying the alternative ADA diagnostic criteria; and (4) further adjusting for smoking and alcohol consumption. The detailed results are presented in Supplementary Tables S3–S7.

First, we removed data with missing values and applied fully adjusted models. We observed a positive correlation between CUN-BAE and prediabetes risk in both male and female groups, consistent with the main study results [male: HR, 95% CI 1.91 (1.59–2.29) for Q2; 2.38 (1.99–2.85) for Q3; 3.22 (2.69–3.87) for Q4; female: HR, 95% CI 1.52 (1.13–2.06) for Q2; 2.36 (1.78–3.14) for Q3; 3.44 (2.57–4.60) for Q4] (Supplementary Table S3). Second, after 1:1 propensity score matching, we identified 15,064 matched pairs in the Q1–Q2 group and 11,119 pairs in the Q3–Q4 group for both male and female participants (Supplementary Table S4). In the fully adjusted models, we found a strong correlation between CUN-BAE and prediabetes risk in both groups [male: HR, 95% CI 1.30 (1.15–1.46) for Q3–Q4 vs. Q1–Q2; female: HR, 95% CI 1.68 (1.36–2.07) for Q3–Q4 vs. Q1–Q2] (Supplementary Table S5). Third, we redefined prediabetes using the ADA (American Diabetes Association) criteria. In the fully adjusted models, we again found a significant association between CUN-BAE and prediabetes [male: HR, 95% CI 1.25 (1.15–1.35) for Q2; 1.52 (1.40–1.64) for Q3; 1.85 (1.71–2.02) for Q4; female: HR, 95% CI 1.37 (1.21–1.55) for Q2; 2.06 (1.82–2.32) for Q3; 2.83 (2.49–3.21) for Q4] (Supplementary Table S6). Lastly, we further incorporated smoking and alcohol consumption variables into the multivariate models, and again found a significant association between CUN-BAE and prediabetes [male: HR, 95% CI 1.86 (1.33–2.61) for Q2; 2.47 (1.77–3.44) for Q3; 3.61 (2.58–5.04) for Q4; female: HR, 95% CI 1.83 (1.00–3.47) for Q2; 3.27 (1.80–5.94) for Q3; 3.93 (2.10–7.35) for Q4] (Supplementary Table S7).

## 4 Discussion

This is the first study investigating longitudinally the association of BF assessed with the CUN-BAE index and the risk of prediabetes in a Chinese population. Even after adjusting for confounding factors, a significant association between CUN-BAE index and prediabetes risk persisted. The fully adjusted RCS regression analysis showed a nonlinear positive correlation between the CUN-BAE index and prediabetes risk in both males and females. In males, prediabetes risk increased significantly when the CUN-BAE index exceeded 22.89, whereas in females, the risk increased significantly when the index surpassed 31.23. Additionally, our study indicates that the CUN-BAE index is a more effective predictor of prediabetes risk compared to the traditional BMI index and the blood biomarkers TG/HDL-c and TyG. Importantly, the positive correlation between the CUN-BAE index and prediabetes risk was consistent across various subgroups, regardless of age, BMI, TG, TC, and hypertension. In men, individuals with higher TG levels or hypertension showed an increased risk of prediabetes as CUN-BAE increased. In women, those in middle age, with obesity (BMI > 28), elevated TG levels, or hypertension, exhibited a higher risk of prediabetes as CUN-BAE increased. These findings suggest that the CUN-BAE index, an alternative marker of body fat, could serve as a valuable tool for predicting prediabetes in primary clinical settings.

Prediabetes is a critical intermediate stage of diabetes, posing significant risks for long-term complications and placing substantial burdens on global healthcare systems (4, 6–9). However, routine blood tests for prediabetes screening are expensive and time-consuming, making body composition assessments a valuable alternative (17, 29). Among the methods for assessing body composition, body mass index (BMI) is widely used owing to its simplicity and accessibility in screening for prediabetes and diabetes (3, 30–32). However, BMI often underestimates the prevalence of obesity, particularly when compared to body fat percentage (BF%), as it does not distinguish fat from muscle, nor does it account for age and gender factors (33, 34). Although DEXA is the gold standard for measuring BF%, its high costs limit its widespread clinical use (19). This highlight the urgent need for alternative indicators that are efficient and practical for clinical use.

The CUN-BAE index has emerged as a valuable tool for estimating BF% in clinical practice, providing a simple and effective alternative to traditional methods (21, 35). Previous studies have shown that higher CUN-BAE values are associated with an increased risk of diabetes (36, 37). Studies have also identified a strong correlation between CUN-BAE and the metabolic syndrome (38, 39). Additionally, CUN-BAE has been found to be a significant predictor of hypertension onset, and is strongly associated with the risk of cardiovascular diseases (24, 36). Although numerous studies have reported the relationship between CUN-BAE and diabetes, there is limited focus on its association with prediabetes. Prediabetes represents an early stage in the development of diabetes, and its underlying mechanisms may differ from those of diabetes. Whether CUN-BAE holds the same predictive value in prediabetes remains to be further investigated. Therefore, our study aims to fill this gap by providing new insights into the relationship between CUN-BAE and prediabetes. Using a nationally representative large-scale dataset with an average follow-up of 3.11 years, our study found a significant association between CUN-BAE and the incidence of prediabetes. The RCS curve analysis revealed a nonlinear positive correlation between CUN-BAE. Additionally, in male, when CUN-BAE > 22.89, and in female, when CUN-BAE > 31.23, the risk of developing prediabetes significantly increased.

Studies conducted in Chinese and Singaporean populations, as well as a prospective 30-year follow-up study in the United States, have reported the relationship between BMI and prediabetes (40, 41). Recent research has also highlighted the close associations of lipid-related composite indices, such as TG/HDL-c, with insulin resistance, metabolic syndrome, and cardiovascular disease risk (42, 43). Additionally, the TyG index, a novel marker reflecting insulin resistance, has shown significant associations with obesity, hypertension, cardiovascular diseases, and prediabetes (44, 45). In our study, we found that the AUC of CUN-BAE for predicting prediabetes was significantly higher than that of BMI, TG/HDL-c, and TyG in both male and female populations (DeLong’s test, all *p* < 0.001), with the following values: males—CUN-BAE: 0.691, TyG: 0.650, BMI: 0.631, TG/HDL-c: 0.598; females—CUN-BAE: 0.759, TyG: 0.715, BMI: 0.707, TG/HDL-c: 0.673. To contextualize the utility of the CUN-BAE, we compare it with three widely used adiposity indices—waist circumference (WC), waist-to-height ratio (WHtR), and Body Adiposity Index (BAI)—as well as the traditional BMI (46–48). These indices differ in their measurement requirements, ability to capture adiposity distribution, and their validity for predicting metabolic risk, particularly in Chinese adults. WC and WHtR are widely used as surrogate markers for visceral fat, which is closely linked to cardiometabolic risk (49, 50). Their main advantage lies in directly assessing central obesity, a key driver of insulin resistance. However, both indices require precise anatomical measurements, which may introduce variability, and they do not account for age- and gender-related differences in fat distribution. BAI, calculated based on hip circumference and height, estimates body fat percentage without the need for weight measurement (51). While it avoids the use of a scale, its correlation with actual body fat may vary across different ethnicities and does not account for age-related changes in fat distribution (52, 53). The key innovation of the CUN-BAE index is its integration of age and sex into the BMI formula, significantly improving the estimation of body fat percentage by considering physiological changes that BMI overlooks. This may explain its superior performance in predicting metabolic conditions, such as prediabetes. However, a common limitation shared by CUN-BAE and all BMI-derived indices is their inability to distinguish between fat mass and lean muscle mass. Therefore, CUN-BAE may still misclassify individuals with high muscle mass, such as athletes or certain labor force groups, as having high adiposity, potentially limiting its accuracy in these specific subgroups. Future studies should directly evaluate the applicability of CUN-BAE in such special populations (e.g., athletes and manual laborers). Furthermore, direct comparisons of the predictive ability of CUN-BAE, WC, WHtR, and BAI for prediabetes within the same general cohort would be valuable in establishing the most effective clinical risk stratification tool.

Subgroup analysis confirmed that the predictive ability of the CUN-BAE index for prediabetes remained robust across different subgroups based on age, BMI, TG, TC, and hypertension. Notably, in both male and female groups, patients with higher TG levels or hypertension experienced a significant increase in prediabetes risk as CUN-BAE values rose. Several factors could explain these differences. The CUN-BAE, as a well-validated estimator of body fat, reflects an increase in body fat as its value rises. Excessive body fat, particularly the accumulation of visceral fat, triggers a series of metabolic changes. TG levels are often concomitant with excessive body fat. TG is a key component of blood lipids, and when lipid metabolism is disrupted, the synthesis of TG increases or its breakdown decreases, leading to elevated blood TG levels (54). Furthermore, high TG levels can influence lipoprotein metabolism, which indirectly affects insulin function (55). In the case of hypertension, prolonged high pressure on the vascular walls results in endothelial cell damage, triggering inflammatory responses and oxidative stress (56, 57). This not only impairs vascular function but also disrupts systemic metabolic regulation. Endothelial dysfunction can affect the transport and action of insulin, preventing it from reaching its target tissues effectively, thereby exacerbating insulin resistance (58). The synergistic effects of CUN-BAE with TG or hypertension contribute to more severe insulin resistance, leading to a marked increase in the incidence of prediabetes as the CUN-BAE index rises (59). Furthermore, in women, we found that the risk of developing prediabetes significantly increased among those in middle age or with obesity (BMI > 28). The potential reasons for this are as follows: First, in middle-aged women, particularly around the peri-menopausal period, significant hormonal changes, especially a decline in estrogen levels, have a substantial impact on metabolism. The reduction in estrogen leads to changes in fat distribution, particularly the accumulation of abdominal and visceral fat (60). The increase in visceral fat not only alters the metabolic characteristics of fat but also promotes the development of insulin resistance, further elevating blood glucose levels and increasing the risk of prediabetes (61). The factors inhibit insulin action, exacerbating insulin resistance, disrupting glucose regulation, and significantly raising the risk of prediabetes. Second, in the obese state, particularly with a higher BMI, the development of insulin resistance is more pronounced in women. As CUN-BAE increases, the rise in body fat, especially visceral fat, leads to the activation of pro-inflammatory factors secreted by adipocytes, such as adipokines and tumor necrosis factor. This activation accelerates the onset of insulin resistance, further increasing the risk of prediabetes (62).

Our study has several notable strengths: (1) It is broadly representative, encompassing multiple regions across China and involving a substantial population sample spanning various age groups. (2) This is the first study to highlight the predictive value of CUN-BAE for prediabetes. Through stratified analysis, we identified specific subpopulations, offering novel approaches for precise prediabetes screening and future diabetes prevention and intervention strategies. (3) The robustness of our findings is further supported by sensitivity and subgroup analyses, which consistently demonstrated a stable positive correlation between CUN-BAE and prediabetes risk across all subgroups, underscoring the reliability of our conclusions.

Nonetheless, we acknowledge certain limitations in our study: (1) We used the World Health Organization’s (WHO) diagnostic criteria for prediabetes, which has a relatively stringent threshold for impaired fasting glucose (IFG), potentially resulting in fewer prediabetes cases being included. However, our sensitivity analysis using the American Diabetes Association’s (ADA) lower IFG threshold (fasting glucose: 5.6–6.9 mmol/L) yielded results consistent with our primary analysis (63). (2) Although prediabetes was defined using fasting glucose according to WHO/ADA criteria, the lack of HbA1c, insulin measurements, and the oral glucose tolerance test (OGTT) likely resulted in an underestimation of its actual prevalence. Future studies should incorporate multiple diagnostic criteria, including OGTT, HbA1c, and insulin measurements, to provide a more accurate and comprehensive assessment of prediabetes. (3) As a retrospective cohort study, residual confounding from unmeasured variables (e.g., dietary habits) remains possible. However, sensitivity analyses adjusting for other lifestyle factors (smoking and alcohol, despite extensive missingness) yielded consistent results, strengthening our confidence that the absence of dietary data did not substantially alter our conclusions. Furthermore, while our exploratory subgroup and interaction analyses revealed interesting patterns, the numerous statistical tests performed increase the potential for Type I error (false positives). We did not adjust for multiple comparisons in these analyses, as they were hypothesis-generating; therefore, these specific results should be interpreted with caution and require validation in independent cohorts. (4) The exclusion of individuals with a follow-up period of less than 2 years, while necessary to ensure adequate time for the development of prediabetes, may have introduced selection bias. However, given the very large initial sample size and the consistent results from our sensitivity analyses, we believe the overall findings remain robust.

## 5 Conclusion

This study revealed a robust and consistent positive nonlinear association between CUN-BAE and the risk of developing prediabetes, underscoring the utility of CUN-BAE as a predictive marker. In male, elevated CUN-BAE, in combination with higher TG levels or the presence of hypertension, was associated with a significantly higher risk of prediabetes. In female, elevated CUN-BAE, combined with factors such as middle age, BMI > 28, higher TG levels, or hypertension, significantly increases the risk of prediabetes. These findings highlight the potential of CUN-BAE to improve the identification of individuals at heightened risk of prediabetes, offering a more nuanced approach than BMI alone.

## Data Availability

The original contributions presented in the study are included in the article/Supplementary material, further inquiries can be directed to the corresponding author.

## Footnotes

^1^ www.Datadryad.org
