# Supplementary material for: The Clínica Universidad de Navarra-Body Adiposity Estimator index is a reliable tool for prediabetes: a multi-center retrospective cohort study
Source: Front Nutr. 2025 Oct 24;12:1685618. doi: 10.3389/fnut.2025.1685618 (PMC12593214; doi:10.3389/fnut.2025.1685618)
Supplement: Supplementary file 1 [file Table_1.DOCX]

Supplementary Material

Figure S1.
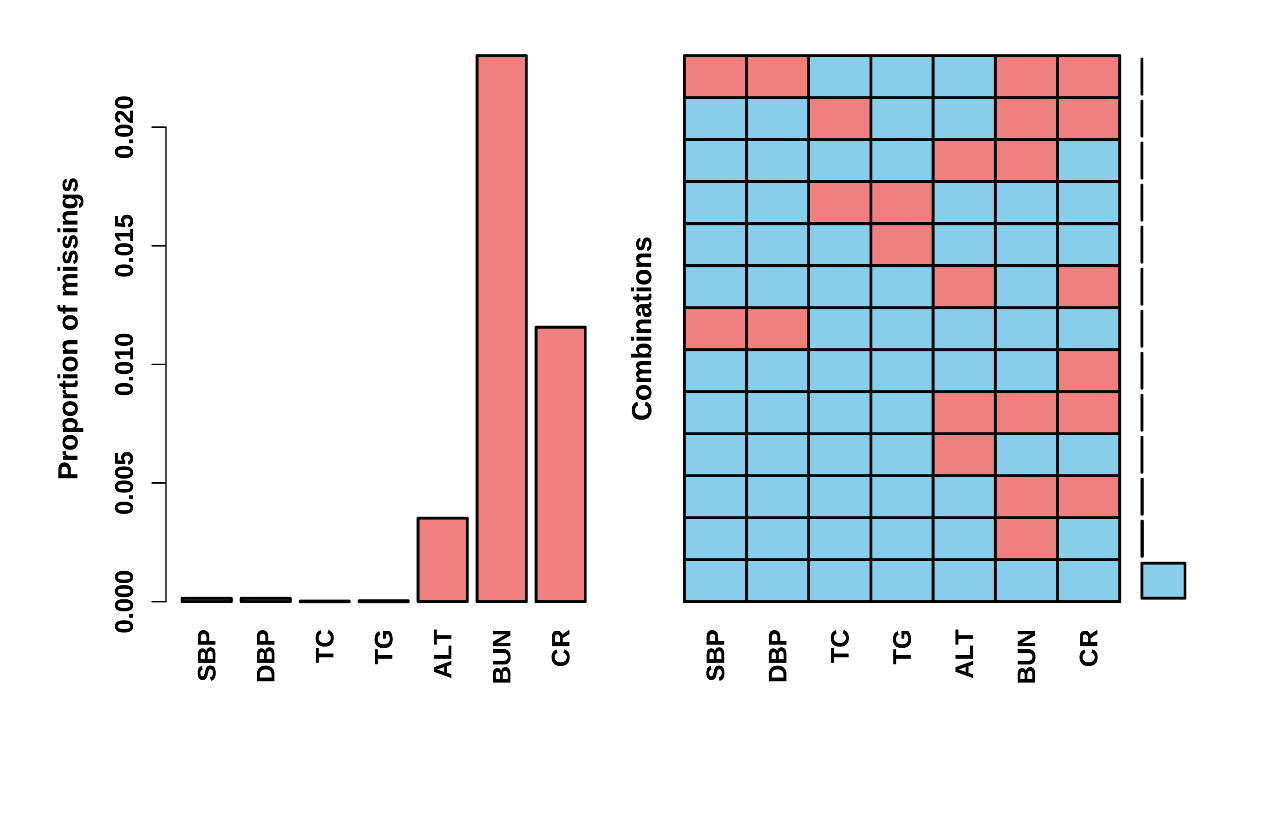
Distribution of variables with missing data.


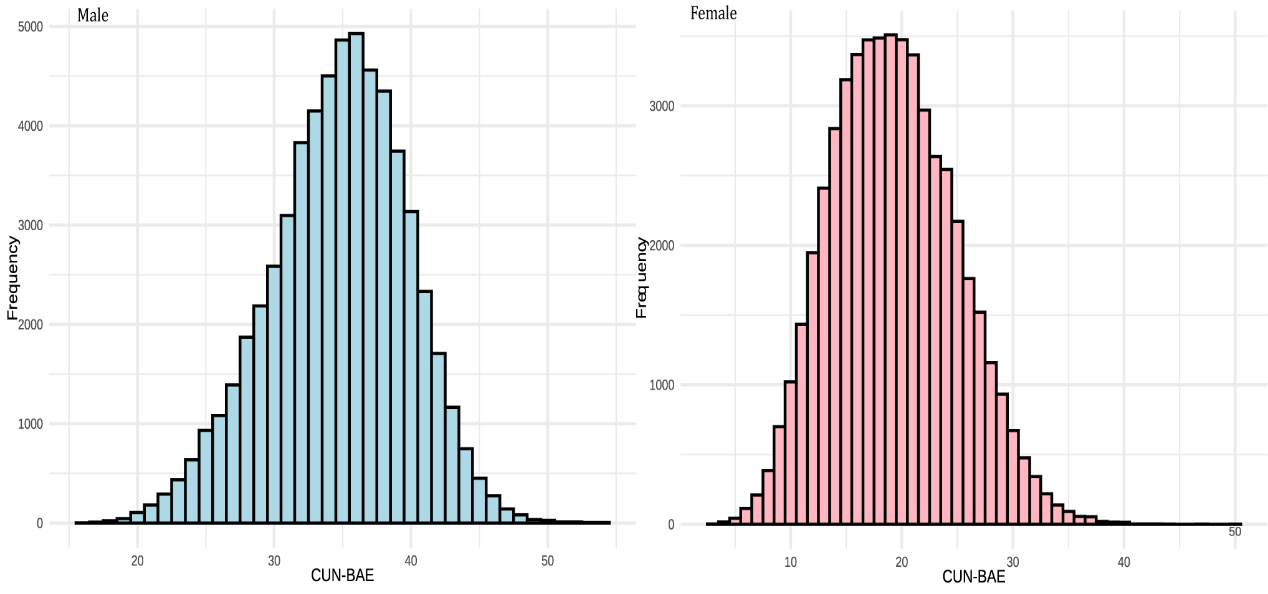
Figure S2. Distribution of CUN-BAE by gender.


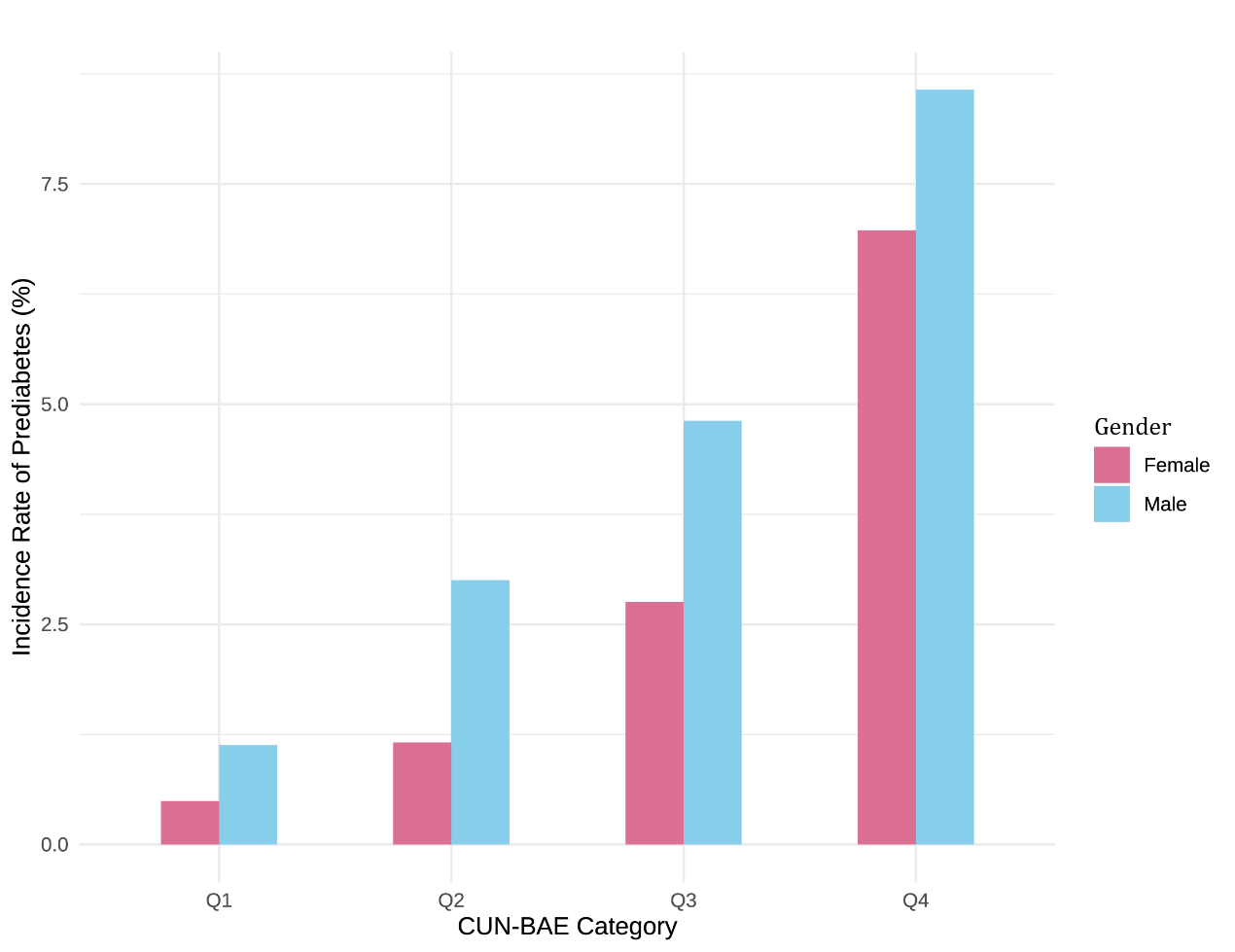


Figure S3. Incidence rates of prediabetes categorized by gender according to CUN-BAE index quartiles. CUN-BAE, Clínica Universidad de Navarra-Body Adiposity Estimator

Table S1. Comparison of Baseline Characteristics Between Included and Excluded Study Populations

| **Characteristics** | **Included Cohort** | **Excluded Cohort** | **p-value** | **SMD** |
| --- | --- | --- | --- | --- |
| n | 112708 | 99125 |  |  |
| Gender (Female) | 52790(46.84) | 52790(46.84) | <0.001 | 0.071 |
| Age, year | 43.64 ± 12.75 | 41.35 (12.30) | <0.001 | 0.183 |
| Height, cm | 166.28 ± 8.32 | 166.60 (8.33) | <0.001 | 0.039 |
| BMI, kg/m2 | 23.26 ± 3.27 | 23.21 (3.43) | <0.001 | 0.015 |
| Family-history |  |  | <0.001 | 0.029 |
| Yes | 2531(2.25) | 1813(1.8) |  |  |
| No | 110177(97.75) | 97312(98.17) |  |  |
| SPB, mmHg | 118.94 ± 16.44 | 119.20(16.30) | <0.001 | 0.016 |
| DBP, mmHg | 74.20 ± 10.89 | 74.15 (10.73) | <0.001 | 0.004 |
| FPG, mmol/L | 4.88 ± 0.55 | 4.95 (0.68) | <0.001 | 0.106 |
| TC, mmol/L | 4.77 ± 0.89 | 4.63 (0.90) | <0.001 | 0.163 |
| TG, mg/dL | 1.35 ± 1.00 | 1.33 (1.07) | <0.001 | 0.019 |
| BUN, mmol/L | 4.67 ± 1.17 | 4.65 (1.21) | <0.001 | 0.016 |
| CR, umol/L | 70.22 ± 15.78 | 69.87 (15.83) | <0.001 | 0.018 |
| ALT, U/L | 23.54 ± 21.64 | 24.42 (22.67) | <0.001 | 0.04 |
| CUN-BAE | 26.78 ± 6.92 | 25.89 ± 7.12 | <0.001 | 0.127 |

BMI, body mass index; SBP, systolic blood pressure; DSP, diastolic blood pressure; FPG, fasting plasma glucose; TC, total cholesterol; TG, triglyceride; BUN, blood urea nitrogen; CR, creatinine; ALT, alanine aminotransferase; CUN-BAE, Clínica Universidad de Navarra-Body Adiposity Estimator.

Table S2. Baseline characteristics of study participants according to CUN-BAE index quartiles

| **Characteristics** | **Overall** | **Quartiles of the CUN-BAE** | | | |  |
| --- | --- | --- | --- | --- | --- | --- |
|  |  | **Quartile 1** | **Quartile 2** | **Quartile 3** | **Quartile 4** | **p-value** |
| n | 112708 | 28179 | 28182 | 28156 | 28191 |  |
| Gender (Female) | 52790(46.84) | 28104(99.73) | 21522(76.37) | 2985(10.60) | 179(0.63) | <0.001 |
| Age, year | 43.64 ± 12.75 | 36.82 ± 8.05 | 45.89 ± 12.91 | 42.27 ± 12.02 | 49.56 ± 13.62 | <0.001 |
| Height, cm | 166.28 ± 8.32 | 161.20 ± 5.45 | 162.42 ± 8.23 | 170.64 ± 7.60 | 170.87 ± 6.40 | <0.001 |
| BMI, kg/m2 | 23.26 ± 3.27 | 20.06 ± 1.55 | 22.82 ± 2.47 | 23.44 ± 2.35 | 26.72 ± 2.49 | <0.001 |
| Smoking status |  |  |  |  |  | <0.001 |
| Current | 6270(19.95) | 10(0.16) | 557(8.23) | 2417(26.02) | 3286(35.70) |  |
| Former | 1263(4.02) | 3(0.05) | 138(2.04) | 537 5.78) | 585(6.36) |  |
| Never | 23902(76.04) | 6163(99.79) | 6071(89.73) | 6334(68.20) | 5334(57.95) |  |
| Drinking status |  |  |  |  |  | <0.001 |
| Current | 798(2.54) | 10(0.16) | 49(0.72) | 275(2.96) | 464(5.04) |  |
| Former | 5292(16.83) | 150(2.43) | 645(9.53) | 2212(23.82) | 2285(24.82) |  |
| Never | 25345(80.63) | 6016(97.41) | 6072(89.74) | 6801(73.22) | 6456(70.14) |  |
| Family-history |  |  |  |  |  | <0.001 |
| Yes | 2531(2.25) | 777(2.76) | 781(2.77) | 511(1.81) | 462(1.64) |  |
| No | 110177(97.75) | 27402(97.24) | 27401(97.23) | 27645(98.19) | 27729(98.36) |  |
| SPB, mmHg | 118.94 ± 16.44 | 108.94 ± 12.49 | 118.88 ± 16.28 | 120.77 ± 15.01 | 127.17 ± 16.25 | <0.001 |
| DBP, mmHg | 74.20 ± 10.89 | 68.44 ± 8.96 | 73.39 ± 10.31 | 75.09 ± 9.99 | 79.87 ± 11.01 | <0.001 |
| FPG, mmol/L | 4.88 ± 0.55 | 4.73 ± 0.51 | 4.90 ± 0.53 | 4.88 ± 0.55 | 5.02 ± 0.55 | <0.001 |
| TC, mmol/L | 4.77 ± 0.89 | 4.54 ± 0.82 | 4.83 ± 0.94 | 4.77 ± 0.87 | 4.96 ± 0.89 | <0.001 |
| TG, mg/dL | 1.35 ± 1.00 | 0.84 ± 0.46 | 1.20 ± 0.76 | 1.45 ± 0.97 | 1.90 ± 1.30 | <0.001 |
| BUN, mmol/L | 4.67 ± 1.17 | 4.19 ± 1.05 | 4.60 ± 1.15 | 4.85 ± 1.14 | 5.02 ± 1.18 | <0.001 |
| HDL-c, mmol/L | 1.37 ± 0.31 | 1.52 ± 0.31 | 1.42 ± 0.29 | 1.32 ± 0.28 | 1.25 ± 0.27 | <0.001 |
| LDL-c, mg/dL | 2.76 ± 0.68 | 2.57 ± 0.62 | 2.80 ± 0.71 | 2.78 ± 0.66 | 2.89 ± 0.68 | <0.001 |
| CR, umol/L | 70.22 ± 15.78 | 57.51 ± 9.38 | 63.91 ± 13.49 | 78.28 ± 13.43 | 81.00 ± 12.77 | <0.001 |
| ALT, U/L | 23.54 ± 21.64 | 14.84 ± 18.53 | 19.24 ± 14.93 | 26.32 ± 20.63 | 33.73 ± 25.98 | <0.001 |
| CUN-BAE | 26.78 ± 6.92 | 15.03 ± 3.03 | 23.76 ± 2.58 | 32.23 ± 2.01 | 38.96 ± 2.63 | <0.001 |

BMI, body mass index; SBP, systolic blood pressure; DSP, diastolic blood pressure; FPG, fasting plasma glucose; TC, total cholesterol; TG, triglyceride; BUN, blood urea nitrogen; HDL-c, high-density lipoprotein cholesterol; LDL-c, low-density lipoprotein cholesterol; CR, creatinine; ALT, alanine aminotransferase; CUN-BAE, Clínica Universidad de Navarra-Body Adiposity Estimator.

Table S3. Association between the baseline CUN-BAE index and incident prediabetes among male and female after excluding individuals with any missing value

| **CUN-BAE** | **crude model** | | **Model 1** | | **Model 2** | | **Model 3** | |
| --- | --- | --- | --- | --- | --- | --- | --- | --- |
|  | **HR (95%CI)** | ***P*** | **HR (95%CI)** | ***P*** | **HR (95%CI)** | ***P*** | **HR (95%CI)** | ***P*** |
| **Male** |  |  |  |  |  |  |  |  |
| Quartiles |  |  |  |  |  |  |  |  |
| Quartiles 1 | Reference |  | Reference |  | Reference |  | Reference |  |
| Quartiles 2 | 2.51(2.10,3.01) | <0.001 | 2.08(1.74,2.49) | <0.001 | 1.99(1.66,2.39) | <0.001 | 1.67(1.39,2.00) | <0.001 |
| Quartiles 3 | 4.01(3.39,4.75) | <0.001 | 2.84(2.38,3.38) | <0.001 | 2.58(2.17,3.08) | <0.001 | 1.93(1.61,2.31) | <0.001 |
| Quartiles 4 | 7.09(6.03,8.34) | <0.001 | 4.29(3.61,5.10) | <0.001 | 3.59(3.02,4.28) | <0.001 | 2.36(1.97,2.84) | <0.001 |
| p for trend |  | <0.001 |  | <0.001 |  | <0.001 |  | <0.001 |
| Per 1 SD increase | 1.96(1.88,2.05) | <0.001 | 1.67(1.59,1.75) | <0.001 | 1.54(1.46,1.62) | <0.001 | 1.33(1.26,1.41) | <0.001 |
| **Female** |  |  |  |  |  |  |  |  |
| Quartiles |  |  |  |  |  |  |  |  |
| Quartiles 1 | Reference |  | Reference |  | Reference |  | Reference |  |
| Quartiles 2 | 2.24(1.66, 3.01) | <0.001 | 1.76(1.31,2.38) | <0.001 | 1.82(1.35,2.45) | <0.001 | 1.50(1.11,2.03) | 0.01 |
| Quartiles 3 | 5.40(4.12, 7.08) | <0.001 | 3.29(2.49,4.34) | <0.001 | 3.33(2.52,4.40) | <0.001 | 2.37(1.79,3.14) | <0.001 |
| Quartiles 4 | 14.51(11.21,18.77) | <0.001 | 6.44(4.87,8.51) | <0.001 | 5.88(4.44,7.80) | <0.001 | 3.44(2.58,4.60) | <0.001 |
| p for trend |  | <0.001 |  | <0.001 |  | <0.001 |  | <0.001 |
| Per 1 SD increase | 2.13(2.04,2.22) | <0.001 | 1.73(1.64,1.83) | <0.001 | 1.62(1.53,1.72) | <0.001 | 1.39(1.31,1.48) | <0.001 |

Crude model, unadjusted; Model 1, adjusted for age, height, family history; Model 2, adjusted for age, height, family history, SBP, DBP; Model 3, adjusted for adjusted for age, height, family history, SBP, DBP, HDL, LDL, ALT, BUN, CR, TG.

Abbreviations: CUN-BAE, Clínica Universidad de Navarra-Body Adiposity Estimator; HR, heart rate; CI, confidence interval; SD, standard deviation.

Table S4. Baseline characteristics before and after 1:1 propensity score matching among male and female

| **Covariates** | **Before Matching** | | |  | **After Matching** | | |
| --- | --- | --- | --- | --- | --- | --- | --- |
|  | **Q1-Q2** | **Q3-Q4** | **SMD** |  | **Q1-Q2** | **Q3-Q4** | **SMD** |
| **Male** |  |  |  |  |  |  |  |
| n | 29454 | 29247 |  |  | 15064 | 15064 |  |
| Age, years | 36.00 (32.00-44.00) | 48.00 (38.00-59.00) | 0.895 |  | 41.00 (34.00-50.00) | 41.00 (34.00-50.00) | 0.013 |
| Height, cm | 172.00 (168.50-176.50) | 171.00 (167.00-175.00) | 0.232 |  | 172.00 (168.00-176.00) | 172.00 (168.00-176.00) | 0.009 |
| Family-history |  |  | 0.005 |  |  |  | 0.003 |
| Yes | 468 (1.59%) | 484 (1.65%) |  |  | 268 (1.78%) | 263 (1.75%) |  |
| No | 28986 (98.41%) | 28763 (98.35%) |  |  | 14796 (98.22%) | 14801 (98.25%) |  |
| SPB, mmHg | 118.00 (109.00-127.00) | 126.00 (116.00-136.00) | 0.567 |  | 121.00 (111.00-130.00) | 121.00 (112.00-130.00) | 0.016 |
| DBP, mmHg | 73.00 (67.00-80.00) | 79.00 (72.00-87.00) | 0.576 |  | 76.00 (70.00-83.00) | 76.00 (70.00-83.00) | 0.006 |
| TG, mg/dL | 1.10 (0.80-1.56) | 1.59 (1.10-2.28) | 0.539 |  | 1.31 (0.93-1.88) | 1.40 (1.00-1.95) | 0.021 |
| BUN, mmol/L | 4.70 (4.03-5.50) | 4.90 (4.20-5.70) | 0.170 |  | 4.79 (4.10-5.57) | 4.80 (4.10-5.59) | 0.004 |
| HDL-c, mmol/L | 1.31 (1.14-1.50) | 1.23 (1.06-1.43) | 0.278 |  | 1.26 (1.09-1.45) | 1.26 (1.09-1.46) | 0.011 |
| LDL-c, mg/dL | 2.62 (2.23-3.05) | 2.83 (2.41-3.29) | 0.306 |  | 2.76 (2.35-3.21) | 2.76 (2.36-3.19) | 0.010 |
| CCR, umol/L | 79.60 (72.50-87.00) | 80.00 (72.70-88.40) | 0.080 |  | 79.70 (72.40-87.30) | 79.90 (72.40-87.60) | 0.004 |
| ALT, U/L | 20.00 (15.00-28.40) | 26.90 (19.00-39.00) | 0.385 |  | 23.00 (16.60-34.00) | 26.00 (19.00-36.30) | 0.056 |
| **Female** |  |  |  |  |  |  |  |
| n | 25612 | 25446 |  |  | 11119 | 11119 |  |
| Age, years | 35.00 (31.00-41.00) | 50.00 (41.00-59.00) | 1.345 |  | 40.00 (35.00-46.00) | 40.00 (35.00-47.00) | 0.031 |
| Height, cm | 161.00 (157.50-165.00) | 159.00 (155.00-163.00) | 0.412 |  | 160.00 (157.00-164.00) | 160.00 (156.70-164.00) | 0.008 |
| Family-history |  |  | 0.026 |  |  |  | 0.003 |
| Yes | 711 (2.78%) | 821 (3.23%) |  |  | 409 (3.68%) | 416 (3.74%) |  |
| No | 24901 (97.22%) | 24625 (96.77%) |  |  | 10710 (96.32%) | 10703 (96.26%) |  |
| SPB, mmHg | 107.00 (100.00-116.00) | 119.00 (108.00-132.00) | 0.806 |  | 111.00 (103.00-120.00) | 111.00 (103.00-120.00) | 0.009 |
| DBP, mmHg | 67.00 (62.00-73.00) | 73.00 (66.00-81.00) | 0.615 |  | 70.00 (64.00-76.00) | 70.00 (64.00-76.00) | 0.006 |
| TG, mg/dL | 0.74 (0.57-0.99) | 1.10 (0.79-1.59) | 0.702 |  | 0.85 (0.62-1.13) | 0.87 (0.65-1.19) | 0.027 |
| BUN, mmol/L | 4.70 (4.03-5.50) | 4.90 (4.20-5.70) | 0.170 |  | 4.15 (3.50-4.90) | 4.17 (3.53-4.92) | 0.002 |
| HDL-c, mmol/L | 1.31 (1.14-1.50) | 1.23 (1.06-1.43) | 0.278 |  | 1.42 (1.25-1.63) | 1.43 (1.26-1.63) | 0.009 |
| LDL-c, mg/dL | 2.62 (2.23-3.05) | 2.83 (2.41-3.29) | 0.306 |  | 2.60 (2.22-3.04) | 2.62 (2.26-3.04) | 0.019 |
| CR, umol/L | 79.60 (72.50-87.00) | 80.00 (72.70-88.40) | 0.080 |  | 57.00 (51.40-62.90) | 57.00 (51.50-63.00) | 0.015 |
| ALT, U/L | 20.00 (15.00-28.40) | 26.90 (19.00-39.00) | 0.385 |  | 13.00 (10.45-17.20) | 14.00 (11.00-19.00) | 0.033 |

Variables are presented as mean ± SD or n (%).SMD, standardized mean differenc

Table S5. Association between the baseline CUN-BAE index and incident prediabetes among male and female after 1:1 propensity score matching

| **CUN-BAE** | **crude model** | | **Model 1** | | **Model 2** | | **Model 3** | |  |
| --- | --- | --- | --- | --- | --- | --- | --- | --- | --- |
|  | **HR (95%CI)** | ***P*** | **HR (95%CI)** | ***P*** | **HR (95%CI)** | ***P*** | **HR (95%CI)** | ***P*** |  |
| **Male** |  |  |  |  |  |  |  |  | |
| Quartiles |  |  |  |  |  |  |  |  | |
| Q1-Q2 | Reference |  | Reference |  | Reference |  | Reference |  | |
| Q3-Q4 | 1.38(1.22,1.55) | <0.001 | 1.35(1.20,1.52) | <0.001 | 1.35(1.20,1.52) | <0.001 | 1.32(1.17,1.49) | <0.001 | |
| Per 1 SD increase | 1.52(1.41,1.64) | <0.001 | 1.45(1.34,1.57) | <0.001 | 1.401.30,1.51) | <0.001 | 1.33(1.23,1.44) | <0.001 | |
| **Female** |  |  |  |  |  |  |  |  | |
| Quartiles |  |  |  |  |  |  |  |  | |
| Q1-Q2 | Reference |  | Reference |  | Reference |  | Reference |  | |
| Q3-Q4 | 1.7(1.38,2.09) | <0.001 | 1.66(1.34,2.04) | <0.001 | 1.65(1.34,2.03) | <0.001 | 1.68(1.36, 2.07) | <0.001 | |
| Per 1 SD increase | 1.64(1.47,1.82) | <0.001 | 1.58(1.41,1.76) | <0.001 | 1.49(1.34,1.67) | <0.001 | 1.42(1.27, 1.58) | <0.001 | |

Crude model, unadjusted; Model 1, adjusted for age, height, family history; Model 2, adjusted for age, height, family history, SBP, DBP; Model 3, adjusted for adjusted for age, height, family history, SBP, DBP, HDL, LDL, ALT, BUN, CR, TG.

CUN-BAE, Clínica Universidad de Navarra-Body Adiposity Estimator; HR, heart rate; CI, confidence interval; SD, standard deviation.

Table S6. Association between the baseline CUN-BAE index and incident prediabetes among male and female using ADA criteria

| **CUN-BAE** | **crude model** | | **Model 1** | | **Model 2** | | **Model 3** | |
| --- | --- | --- | --- | --- | --- | --- | --- | --- |
|  | **HR (95%CI)** | ***P*** | **HR (95%CI)** | ***P*** | **HR (95%CI)** | ***P*** | **HR (95%CI)** | ***P*** |
| **Male** |  |  |  |  |  |  |  |  |
| **Quartiles** |  |  |  |  |  |  |  |  |
| Quartiles 1 | Reference |  | Reference |  | Reference |  | Reference |  |
| Quartiles 2 | 1.40(1.30,1.52) | <0.001 | 1.27(1.18,1.38) | <0.001 | 1.25(1.15,1.35) | <0.001 | 1.25(1.15,1.35) | <0.001 |
| Quartiles 3 | 1.89(1.75,2.03) | <0.001 | 1.58(1.46,1.70) | <0.001 | 1.50(1.39,1.62) | <0.001 | 1.50(1.38,1.62) | <0.001 |
| Quartiles 4 | 2.57(2.40,2.76) | <0.001 | 1.98(1.84,2.14) | <0.001 | 1.78(1.65,1.93) | <0.001 | 1.76(1.62,1.92) | <0.001 |
| p for trend |  | <0.001 |  | <0.001 |  | <0.001 |  | <0.001 |
| Per 1 SD increase | 1.42(1.39,1.46) | <0.001 | 1.30(1.26,1.33) | <0.001 | 1.24(1.20,1.27) | <0.001 | 1.23(1.19,1.27) | <0.001 |
| **Female** |  |  |  |  |  |  |  |  |
| **Quartiles** |  |  |  |  |  |  |  |  |
| Quartiles 1 | Reference |  | Reference |  | Reference |  | Reference |  |
| Quartiles 2 | 1.59(1.41,1.80) | <0.001 | 1.36(1.21,1.54) | <0.001 | 1.39(1.23,1.57) | <0.001 | 1.38(1.22,1.57) | 0.01 |
| Quartiles 3 | 2.88(2.58,3.22) | <0.001 | 2.07(1.84,2.33) | <0.001 | 2.08(1.85,2.33) | <0.001 | 2.05(1.82,2.31) | <0.001 |
| Quartiles 4 | 5.53(4.98,6.14) | <0.001 | 3.20(2.84,3.61) | <0.001 | 2.92(2.59,3.30) | <0.001 | 2.79(2.46,3.16) | <0.001 |
| p for trend |  | <0.001 |  | <0.001 |  | <0.001 |  | <0.001 |
| Per 1 SD increase | 1.73(1.68,1.77) | <0.001 | 1.46(1.42,1.51) | <0.001 | 1.39(1.34,1.43) | <0.001 | 1.36(1.31,1.41) | <0.001 |

Crude model, unadjusted; Model 1, adjusted for age, height, family history; Model 2, adjusted for age, height, family history, SBP, DBP; Model 3, adjusted for adjusted for age, height, family history, SBP, DBP, HDL, LDL, ALT, BUN, CR, TG, FPG.CUN-BAE, Clínica Universidad de Navarra-Body Adiposity Estimator; HR, heart rate; CI, confidence interval; SD, standard deviation.

Table S7 Association between the baseline CUN-BAE index and incident prediabetes among male and female after excluding individuals with missing smoking and drinking data.

| **CUN-BAE** | **crude model** | | **Model 1** | | **Model 2** | | **Model 3** | |
| --- | --- | --- | --- | --- | --- | --- | --- | --- |
|  | HR (95%CI) | *P* | HR (95%CI) | *P* | HR (95%CI) | *P* | HR (95%CI) | *P* |
| **Male(n = 20406)** |  | | | | | | | |
| **Quartiles** |  |  |  |  |  |  |  |  |
| Quartiles 1 | Reference |  | Reference |  | Reference |  | Reference |  |
| Quartiles 2 | 2.62(1.88,3.65) | <0.001 | 2.24(1.60,3.13) | <0.001 | 2.11(1.51,2.94) | <0.001 | 1.86(1.33,2.61) | <0.001 |
| Quartiles 3 | 4.30(3.15,5.88) | <0.001 | 3.23(2.35,4.45) | <0.001 | 2.89(2.09,3.99) | <0.001 | 2.47(1.77,3.44) | <0.001 |
| Quartiles 4 | 8.17(6.05,11.01) | <0.001 | 5.37(3.92,7.36) | <0.001 | 4.67(3.24,6.15) | <0.001 | 3.61(2.58,5.04) | <0.001 |
| p for trend |  | <0.001 |  | <0.001 |  | <0.001 |  | <0.001 |
| Per 1 SD increase | 1.99(1.86,2.15) | <0.001 | 1.77(1.63,1.92) | <0.001 | 1.64(1.51,1.79) | <0.001 | 1.52(1.39,1.68) | <0.001 |
| **Female(n = 11172)** |  |  |  |  |  |  |  |  |
| **Quartiles** |  |  |  |  |  |  |  |  |
| Quartiles 1 | Reference |  | Reference |  | Reference |  | Reference |  |
| Quartiles 2 | 2.18(1.16, 4.08) | 0.015 | 1.84(1.01,3.50) | 0.040 | 1.86(1.02,3.52) | 0.033 | 1.83(1.00,3.47) | 0.048 |
| Quartiles 3 | 5.20( 2.94, 9.20) | <0.001 | 3.42(1.90,6.19) | <0.001 | 3.44(1.90,6.22) | <0.001 | 3.27(1.80,5.94) | <0.001 |
| Quartiles 4 | 10.20(5.89,17.67) | <0.001 | 5.14(2.80,9.41) | <0.001 | 4.66(2.53,8.58) | <0.001 | 3.93(2.10,7.35) | <0.001 |
| p for trend |  | <0.001 |  | <0.001 |  | <0.001 |  | <0.001 |
| Per 1 SD increase | 2.14(1.90,2.41) | <0.001 | 1.73(1.48,2.00) | <0.001 | 1.62(1.39,1.89) | <0.001 | 1.52(1.29,1.80) | <0.001 |

Crude model, unadjusted; Model 1, adjusted for age, height, family history; Model 2, adjusted for age, height, family history, SBP, DBP; Model 3, adjusted for adjusted for age, height, family history, SBP, DBP, HDL, LDL, ALT, BUN, CR, TG, FPG, smoking status, drinking status. CUN-BAE, Clínica Universidad de Navarra-Body Adiposity Estimator; HR, heart rate; CI, confidence interval; SD, standard deviation.
